# Biocompatible Fe_3_O_4_ Increases the Efficacy of Amoxicillin Delivery against Gram-Positive and Gram-Negative Bacteria

**DOI:** 10.3390/molecules19045013

**Published:** 2014-04-22

**Authors:** Alexandru Mihai Grumezescu, Monica Cartelle Gestal, Alina Maria Holban, Valentina Grumezescu, Bogdan Ștefan Vasile, Laurențiu Mogoantă, Florin Iordache, Coralia Bleotu, George Dan Mogoșanu

**Affiliations:** 1 Department of Science and Engineering of Oxide Materials and Nanomaterials, Faculty of Applied Chemistry and Materials Science, Politehnica University of Bucharest, Polizu Street no 1-7, 011061 Bucharest, Romania; E-Mails: grumezescu@yahoo.com (A.M.G.); valentina_grumezescu@yahoo.com (V.G.); bogdan.vasile@upb.ro (B.S.V.); 2 School of Medicine, Faculty of Public Health, SENESCYT 9 de Octubre N22-64 y Ramírez Dávalos - Casa Patrimonial, 170517 Quito, Ecuador; 3 Microbiology Immunology Department, Faculty of Biology, University of Bucharest, Aleea Portocalelor no 1-3, 060101 Bucharest, Romania; E-Mail: alina_m_h@yahoo.com; 4 Research Center for Microscopic Morphology and Immunology, University of Medicine and Pharmacy of Craiova, 2 Petru Rareş Street, 200349 Craiova, Romania; E-Mail: editor@rjme.ro; 5 Institute of Cellular Biology and Pathology of Romanian Academy, “Nicolae Simionescu”, Department of Fetal and Adult Stem Cell Therapy, 8, B.P. Hasdeu, 050568 Bucharest, Romania; E-Mail: floriniordache84@yahoo.com; 6 Stefan S. Nicolau Institute of Virology, 285 Mihai Bravu, 030304 Bucharest, Romania; E-Mail: c_bleotu@yahoo.com; 7 Department of Pharmacognosy & Phytotherapy, Faculty of Pharmacy, University of Medicine and Pharmacy of Craiova, 2 Petru Rareş Street, 200349 Craiova, Romania; E-Mail: george.mogosanu@umfcv.ro

**Keywords:** magnetite bio-active nanostructure, amoxicillin, MIC, *S. aureus*, *E. coli*

## Abstract

This paper reports the synthesis and characterization of amoxicillin- functionalized magnetite nanostructures (Fe_3_O_4_@AMO), revealing and discussing several biomedical applications of these nanomaterials. Our results proved that 10 nm Fe_3_O_4_@AMO nanoparticles does not alter the normal cell cycle progression of cultured diploid cells, and an *in vivo* murine model confirms that the nanostructures disperse through the host body and tend to localize in particular sites and organs. The nanoparticles were found clustered especially in the lungs, kidneys and spleen, next to the blood vessels at this level, while being totally absent in the brain and liver, suggesting that they are circulated through the blood flow and have low toxicity. Fe_3_O_4_@AMO has the ability to be easily circulated through the body and optimizations may be done so these nanostructures cluster to a specific target region. Functionalized magnetite nanostructures proved a great antimicrobial effect, being active against both the Gram positive pathogen *S. aureus* and the Gram negative pathogen *E. coli*. The fabricated nanostructures significantly reduced the minimum inhibitory concentration (MIC) of the active drug. This result has a great practical relevance, since the functionalized nanostructures may be used for decreasing the therapeutic doses which usually manifest great severe side effects, when administrated in high doses. Fe_3_O_4_@AMO represents also a suitable approach for the development of new alternative strategies for improving the activity of therapeutic agents by targeted delivery and controlled release.

## 1. Introduction

The rate of development of bacterial resistance to current antibiotics is significantly higher than the rate at which researchers are producing novel antibiotics [1,2,3]. The most frequently encountered nosocomial pathogen exhibiting resistance and multi drug resistance (MDR) patterns is *Staphylococcus aureus*. This pathogen may be encountered on normal human skin and mucosa and has a great adaptability to antibiotic pressure [4]. Methicillin-resistant *S. aureus* (MRSA) represents one of the major etiology of severe hospital acquired infections and increased mortality in these patients [5].

Another commensal bacterium of humans and animals whose pathogenic variants may cause severe infections, including gastroenteritis, urinary tract infection, meningitis, peritonitis, and septicemia is *Escherichia coli* [6,7]. Surveillance data show that resistance in *E. coli* is consistently high for antimicrobial agents that have been in use for a long time in human and veterinary medicine, but also in food industry [8].

Some pathogens, such as the versatile *Pseudomonas aeruginosa* and *Mycobacterium tuberculosis* also possess a high level of intrinsic resistance, which is attributable to a concerted action of multidrug efflux pumps with chromosomally encoded antibiotic resistance genes and the low permeability of the bacterial cellular envelope [9,10].

Since the development of novel antimicrobial drugs is a very elaborate and costly process, research has focused on alternative methods for fighting infections. Using natural products and compounds may be an efficient approach in combating infections [11,12,13], but our current knowledge fails to ensure the success of a therapy based on these compounds in many conditions. This is because the lack of scientific evidence of the intimate mechanisms of action and their effect and distribution within the host. Another potentially efficient strategy relies on using magnetite nanosystems to improve the efficiency of current antimicrobials with proved effect [14,15,16,17,18,19,20,21,22,23,24,25].

The aim of this study was to synthesize, characterize and test the antimicrobial potentiating effect of a biocompatible Fe_3_O_4_-amoxicillin nanosystem, using both a Gram negative and a Gram positive bacteria model.

## 2. Results and Discussion

**Figure 1:**
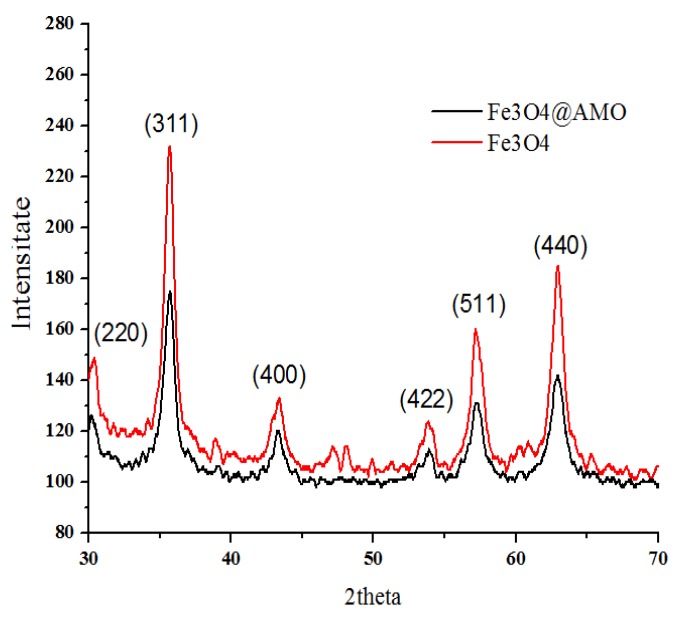
XRD patterns of Fe_3_O_4_@AMO and control Fe_3_O_4__._

The crystalline properties of the prepared nanostructure (Fe3O4@AMO) were investigated by X-Ray diffraction (XRD). The XRD pattern of sample are shown in Figure 1 and all detectable peaks can be assigned to a pure cubic structured Fe_3_O_4_ (JCPDS no. 65-3107) [26]. No additional peaks have been observed indicating the formation of pure and single crystalline phase.

**Figure 2:**
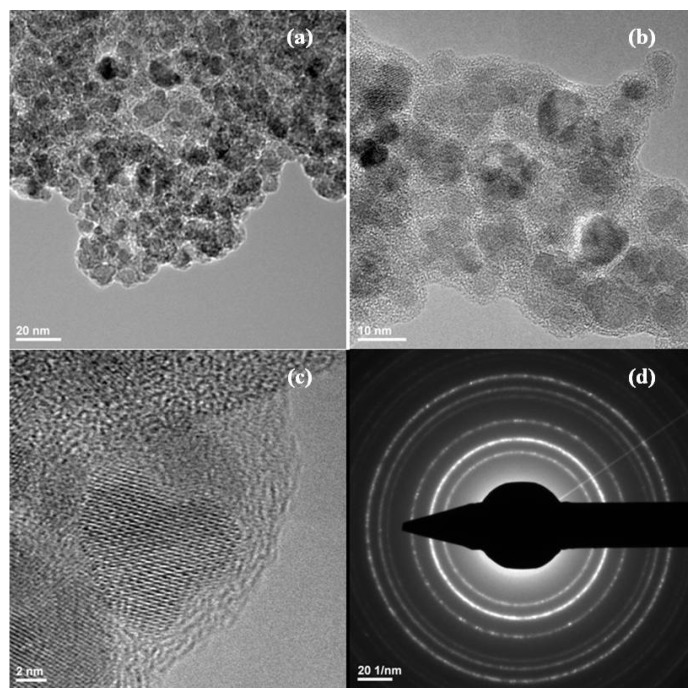
TEM images (**a**,**b**), HR-TEM image (**c**) and SAED pattern (**d**) of magnetite nanoparticles coated with amoxicillin (Fe3O4@AMO).

Transmission electron microscopy allows obtaining information about the sizes and shapes of the prepared nanostructures. Figure 2 present the TEM images of the Fe_3_O_4_@AMO particles from which it can be seen that the crystalline particles are coated with a non-crystalline layer. The size of prepared particles is about 10 nm. No aggregates have been observed, the Fe_3_O_4_@AMO maintaining their nanometric size. The SAED rings, exhibit the high polycrystalline nature of the magnetite without the presence of any other crystalline phases [27].

**Figure 3:**
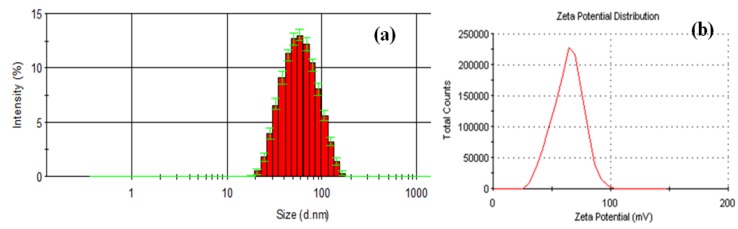
DLS histogram (**a**) and zeta potential distribution (**b**) of the Fe_3_O_4_@AMO nanoparticles.

Figure 3a presents the size distribution histogram of Fe_3_O_4_@AMO. The size distribution at 25 °C showed a hydrodynamic particle size average at 52 nm. Fe_3_O_4_@AMO exhibited a positive zeta potential of about 70 mV (Figure 3b), with a higher colloidal stability, being favorable for the electrostatic interaction with the negatively charged bacterial wall. This fact allows a better release of antibiotics inside the bacterial cell [21].

**Figure 4:**
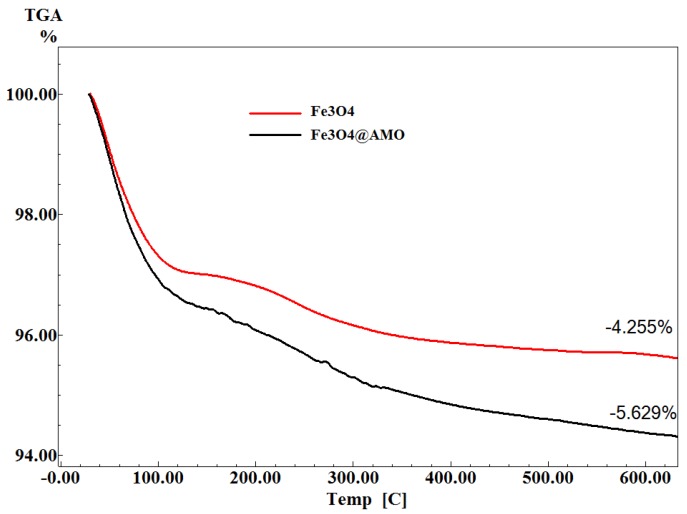
Thermogravimetric curves for magnetite nanoparticles with and without amoxicillin.

The TG analysis of the Fe_3_O_4_@AMO was performed in order to estimate the percent of AMO entrapped on the surface of Fe_3_O_4_ by comparison with the control (Fe_3_O_4_). The results of TG analysis are shown in Figure 4. The weight loss between 25 and 600 °C refers to the evaporation of adsorbed water and due to the decomposition of the physisorption and the chemisorption of the therapeutic agent [28,29]. Therefore, the content of the amoxicillin from Fe_3_O_4_@AMO was about 1.37%.

**Figure 5:**
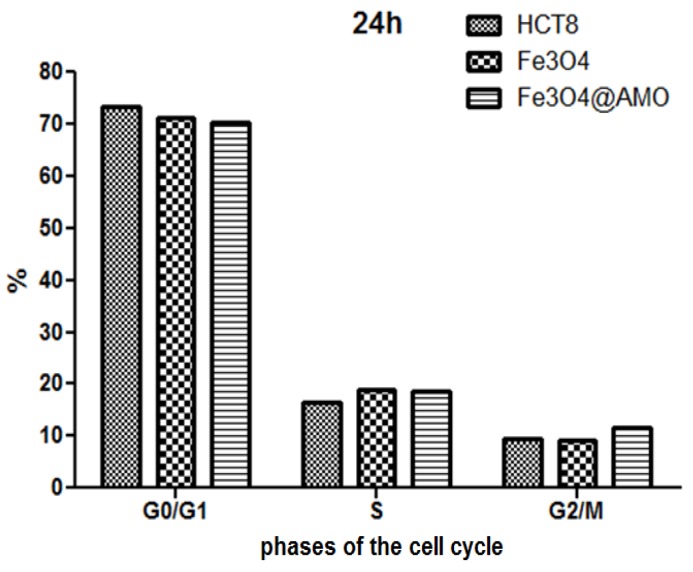
Flow cytometry results revealing the cell cycle progression of HCT8 cultured cells after the treatment for 24 h with the Fe_3_O_4_@AMO nanostructures. K = control HCT8 culture (untreated), Fe_3_O_4_ = HCT8 cultures grown in the presence of 1 mg/mL Fe_3_O_4_; Fe_3_O_4_@AMO = HCT8 cultures grown in the presence of 1 mg/mL Fe_3_O_4_@AMO. *p* < 0.5, based on One Way ANOVA test.

Flow cytometry analysis revealed that the tested nanostructures (Fe_3_O_4_@AMO) have a great biocompatibility *in vitro*, allowing the normal development of cultured eukaryotic cells. The results demonstrated that the Fe_3_O_4_@AMO does not alter the normal progression of the cell cycle in cultured human cells, the percentage of cells encountered in different stages of cell cycle being similar with those obtained for the untreated control (Figure 5).

**Figure 6:**
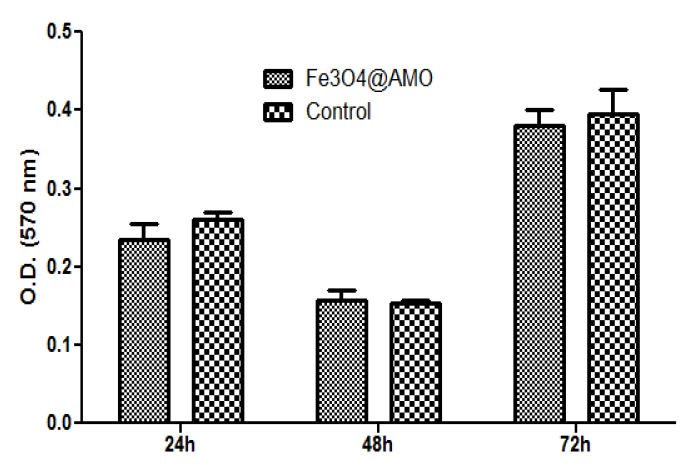
Graphic representation of the MTT results obtained by analyzing endothelial cells grown in the presence of tested Fe_3_O_4_@AMO for 24, 48 and 72 h.

**Figure 7:**
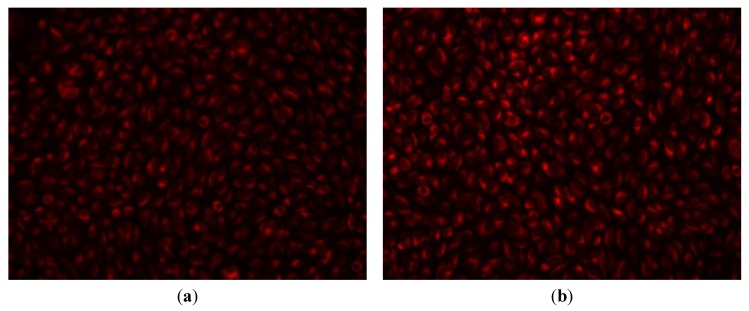
Fluorescence microscopy images of endothelial cells grown in the presence of Fe_3_O_4_@AMO (**a**) and in standard conditions (**b**) at 5 days of incubation.

The flow cytometry results were confirmed by the MTT assay (Figure 6) and microscopy data (Figure 7), which proved that endothelial cells grown in the presence of Fe_3_O_4_@AMO for up to five days have normal behavior and aspect, being similar to untreated control cells.

**Figure 8:**
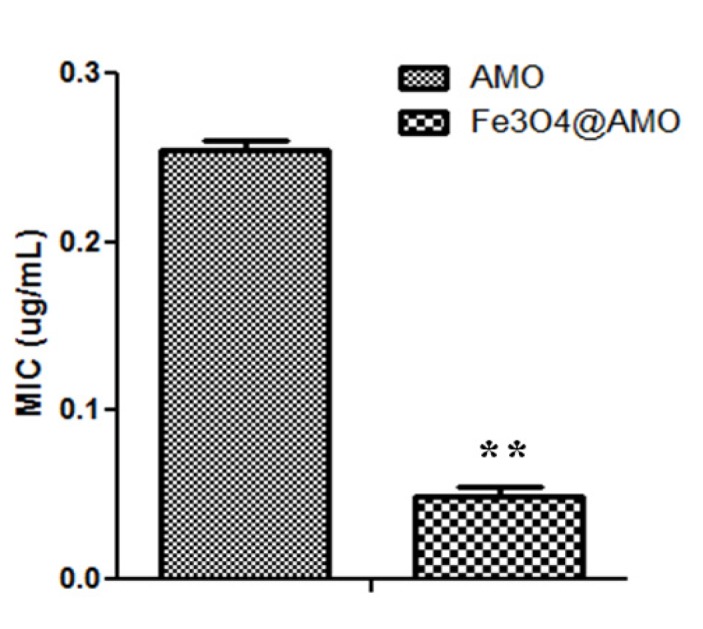
MIC values obtained for the nanoparticles functionalized with antibiotics against *S. aureus* ATCC 29213, as compared with control MIC values, represented by plain antibiotic solutions, ** *p* < 0.05.

**Figure 9:**
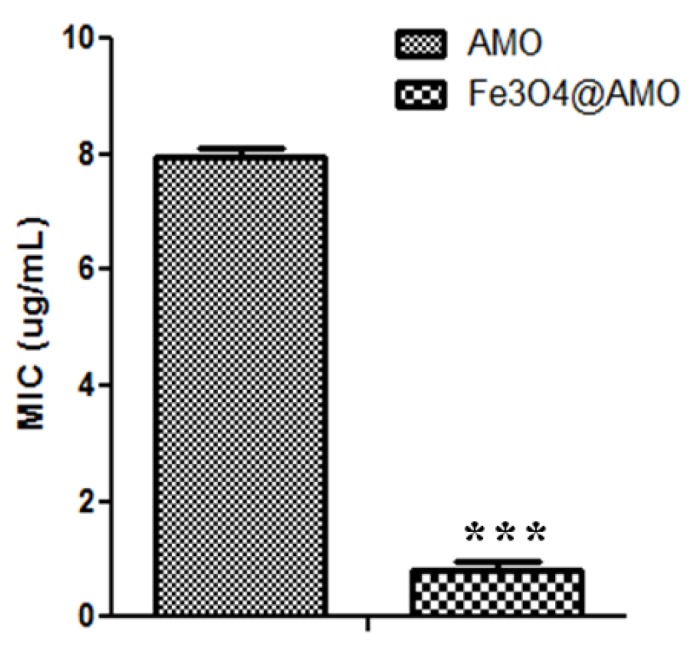
MIC values obtained for the nanoparticles functionalized with antibiotics against *E. coli* ATCC 25922, as compared with control MIC values, represented by plain antibiotic solutions. *** *p* < 0.01.

Despite their low effect on eukaryote cells, functionalized magnetite materials proved a great antimicrobial effect, being active against both the Gram-positive pathogen *S. aureus* and the Gram-negative pathogen *E. coli*. The results demonstrate that the tested nanostructures are very efficient in inhibiting bacteria growth, revealing a very low MIC value. Furthermore, the obtained MIC value for the Fe_3_O_4_@AMO proved to be significantly lower than the one obtained for the plain antibiotic solution (Figure 8 and Figure 9). The fabricated nanomaterials proved to reduce the necessary MIC of amoxicillin to about 3-fold for *S. aureus* and about 4-fold for *E. coli*.

**Figure 10:**
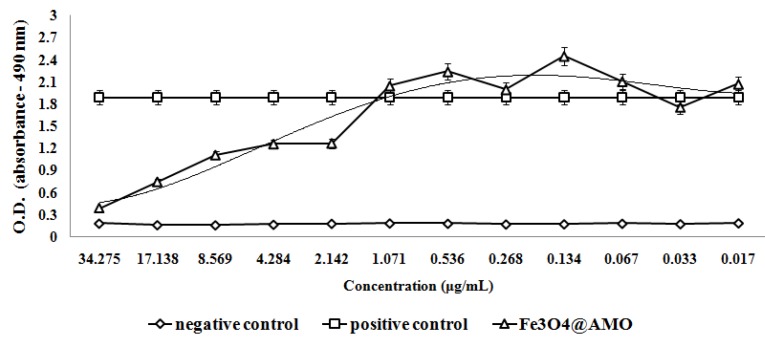
The effect of Fe_3_O_4_@AMO on *S. aureus* adherence at the inert substrata after 24 h of incubation.

**Figure 11:**
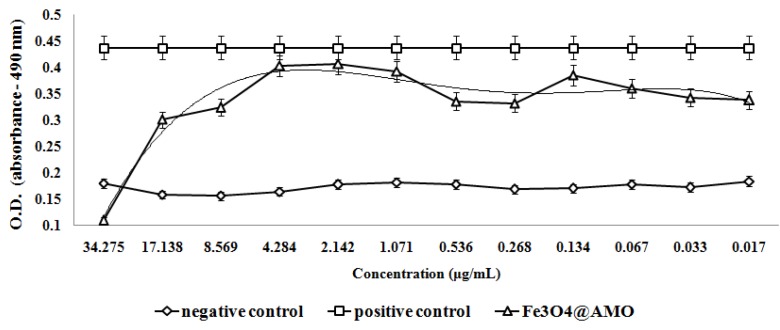
The effect of Fe_3_O_4_@AMO on *E. coli* adherence at the inert substrata after 24 h of incubation.

The antimicrobial assay results demonstrate that these nanostructures also impacts on bacteria adherence. A concentration equal or higher to 2.142 µg/mL proved to be sufficient to inhibit the adherence of *S. aureus* on the bottom of the plate; while the minimum concentration necessary for adherence inhibition in *E. coli* proved to be 0.556 µg/mL (Figure 10 and Figure 11).

This result demonstrates that the nanostructures may be efficiently used as targeted delivery nanoshuttles, and have a great potentiating effect on antibiotics’ activity. The fact that these nanostructures reduce the necessary amount of antibiotic for reaching the MIC effects has a great practical relevance, since it may be used for decreasing the therapeutic doses which usually manifest considerable and severe side effects, when administrated in high amounts. Fe_3_O_4_@AMO also represents a suitable approach for the development of new alternative strategies for improving the activity of therapeutic agents by targeted delivery and controlled release. Therefore, our results contribute to the current knowledge on the field of novel bio-active nanostructured materials and bring new insights within the development of efficient therapeutic strategies by stabilizing and lowering the amount of active compounds used for therapy. This approach may impact on economical, ecological and medical fields because the use of lower amounts of active compounds for treating bacterial infections with the same efficiency lead to decreased costs, lower toxicity and side effects, as well as a lower chemical pollution rate.

**Figure 12:**
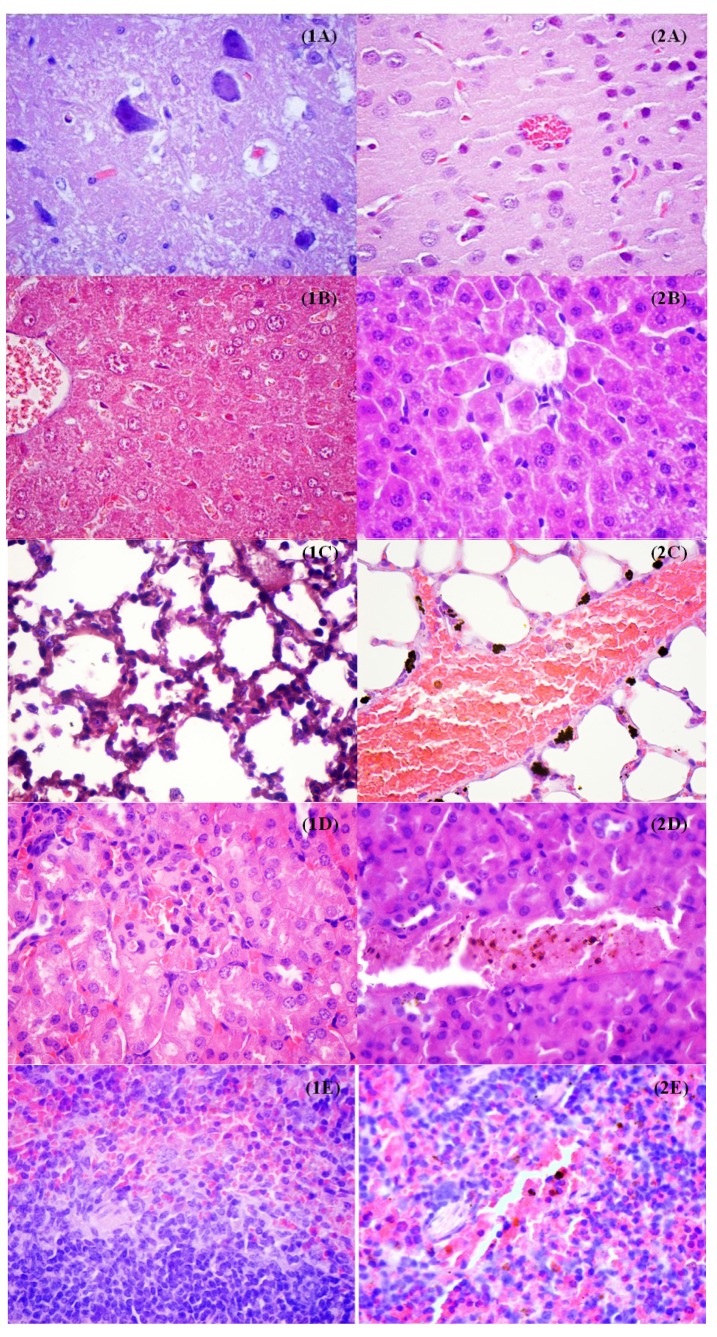
Transversal sections through different mice organs (**A** = brain, **B** = liver, **C** = lung, **D** = kidney, **E** = spleen) in untreated control (1) and after the treatment with the obtained magnetite nanoparticles for 48 h (2); ×400 magnification.

The low cytotoxicity effects proved *in vitro* were confirmed also *in vivo* on a murine model. The results demonstrated that the Fe_3_O_4_@AMO nanostructures disperse through the host body and tend to localize in particular sites and organs. After 48 h of treatment with these dispersible nanoparticles, they were found clustered especially in the lungs, kidneys and spleen, while totally lacking in the brain and liver. The distribution of these nanostructures within the blood irrigated areas of these organs, suggest that they are circulated through the blood flow. This hypothesis is supported by the organ sections images that reveal a preferential localization of tested nanoparticles along the blood vessels (Figure 12). This observation is very useful for the development of therapeutic approaches for systemic and localized infections, because these nanostructures have the ability to be easily circulated and optimizations may be done so these nanostructures cluster to a specific target region. The results also revealed that the analyzed organs tissues are not affected after the treatment with the fabricated nanostructures, they manifesting a normal aspect. However, in the lungs it can be noticed an increased percent of inflammatory cells (as appreciated by comparing treating and untreated mice samples), as neutrophils and macrophages, and many of the observed macrophages contain high amounts of intracellular nanoparticles. The occurrence of an inflammatory process is although normal when injecting a foreign suspension within the body. No lesion typical or suggestive for tubular necrosis and glomerulopathy were observed in the kidneys, as revealed by the analyzed microscopy images obtained from tissue sections (Figure 12D).

## 3. Experimental Section

### 3.1. Synthesis of Magnetite Nanostructures

All chemicals used in the experiments were of analytical reagent grade and were used without further purification. The protocol developed by Grumezescu *et al.* was used as the basic synthesis method of magnetite nanostructures [30,31]. Briefly, amoxicillin (AMO, 100 mg) and NH_4_OH (4 mL, 25%) were mixed in deionized water (400 mL) in order to prepare the alkali solution. FeCl_3_ (0.65 g,) and FeSO_4_ × 7H_2_O (1 g) aqueous solution (100 mL) was added drop by drop into alkali solution (400 mL) under vigorous stirring for 30 min at the room temperature. The pH value was higher than 12. A dark suspension of Fe_3_O_4_@AMO was obtained. In order to purify the prepared nanostructures, Fe_3_O_4_@AMO was separated with a strong NdFeB permanent magnet (100 Kgf), repeatedly washed with deionized water and methanol, and finally dried at room temperature.

### 3.2. Characterization of Magnetite Nanostructures

#### 3.2.1. X-ray Diffraction

X-ray diffraction analysis was performed on a Shimadzu XRD 6000 diffractometer at room temperature. In all the cases, Cu Kα radiation from a Cu X-ray tube (run at 15 mA and 30 kV) was used. The samples were scanned in the Bragg angle 2θ range of 10–80 degree.

#### 3.2.2. Transmission Electron Microscopy

The transmission electron microscopy (TEM) images were obtained on finely powdered samples using a Tecnai™ G2 F30 S-TWIN high resolution transmission electron microscope from FEI Company (Hillsboro, OR, USA) equipped with SAED. The microscope operated in transmission mode at 300 kV with TEM point resolution of 2 Å and line resolution of 1 Å. The microspheres were dispersed into pure ethanol and ultrasonicated for 15 min. After that, diluted sample was poured onto a holey carbon-coated copper grid and left to dry before TEM analysis.

#### 3.2.3. Dynamic Light Scattering (DLS)

Particles size analysis was performed using intensity distribution by dynamic light scattering technique (Zetasizer Nano ZS, Malvern Instruments Ltd., Malvern, UK), at scattering angles of 90° and 25 °C. The average diameters (based on Stokes–Einstein equation) were calculated from three individual measurements. The zeta potential was measured using the Zetasizer Nano ZS.

#### 3.2.4. Thermogravimetric Analysis

The thermogravimetric (TG) analysis of the biocomposite was assessed with a Shimadzu DTG-TA-50H instrument. Samples were screened to 200 mesh prior to analysis, were placed in alumina crucible, and heated with 10 K·min^−1^ from room temperature to 800 °C, under the flow of 20 mL·min^−1^ dried synthetic air (80% N_2_ and 20% O_2_).

#### 3.2.5. Cell Cycle

HCT8 (ECAC90032006) line was cultivated in RPMI 1640 (Gibco, New York, NY, USA) supplemented with 10% heat-inactivated bovine serum and penicillin/streptomycin at 37 °C with 5% CO_2_. HCT8 cell line was treated with 100 µg/mL of the tested nanoparticles suspension, and maintained for 24 h at 37 °C, in 5% CO_2_ and humid atmosphere. After the incubation time, cells were harvested, washed with phosphate buffered saline (PBS) (pH 7.5), fixed in 70% cold ethanol and maintained overnight at −20 °C. Each sample was washed in PBS, treated with 100 µg/mL RNase A for 15 min and stained with 10 µg/mL propidium iodide by incubation at 37 °C for 1 h, following a protocol adapted from [32]. After propidium iodide staining of the cells, the events acquisition was done using an Epics Beckman Coulter flow cytometer. The obtained data were analysed using FlowJo software and expressed as fractions of cells in different cell cycle phases.

#### 3.2.6. Cell Viability

Human endothelial cells (EAhy926 cell line, ATCC, Manassas, VA, USA) were grown in DMEM culture medium containing 10% FBS, and 1% penicillin and neomycin (Sigma Aldrich, St. Louis, MO, USA). For cell proliferation and viability was used CellTiter96 Non-Radioactive Cell Proliferation Assay, (Promega, Madison, WI, USA). Endothelial cells were seeded in 96-well plate at a density of 5 × 10^3^ cells/well, in DMEM medium, supplemented with 10% FBS and incubated with NMS materials for 72 h, while the controls were represented by endothelial cells grown in the same culture conditions, but on bare substrates. Cell proliferation assay was performed in triplicates, according to manufacturer’s guidelines, at different time intervals. Briefly, 15 µL of Promega Kit Solution I was added in each well and incubated for 4 h. Furthermore, 100 µL of Promega Kit Solution II was added in the 96-well plate and incubated for another hour and spectrophotometry mesurements were performed at 570 nm using a Mithras LB 940 spectrophotometer (Berthold Technology, Bad Wildbad, Germany).

RED CMTPX fluorophore (Life Technologies, Invitrogen, Carlsbad, CA, USA) is a cell tracker for long-term tracing of living cells. The RED CMTPX dye was added in the culture medium at a final concentration of 5 µM, incubated 30 min for the dye to penetrate through the cells. Furthermore, the cells were washed with PBS and visualized by fluorescent microscopy. The nuclei were counterstaining with DAPI (1 mg/mL). Living cells tracing in the presence of nanospheres was monitored for 5 days in culture. The micrographs were taken by a digital camera driven by the Axio-Vision 4.6 (Carl Zeiss, Munich, Germany) software.

#### 3.2.7. *In Vivo* Biodistribution

The experimental protocol was applied according with the European Council Directive No. 86/609/24 November 1986, the European Convention on the Protection of Vertebrate Animals (2005) and the Romanian Government Ordinance No. 37/2 February 2002. The mice organs were collected under general anesthesia. Biological material was fixed, directly after the sampling, in 10% buffered neutral formalin, for 72 h, at room temperature, and then processed for routinely histological paraffin embedding technique. For the histological study of nanoparticles, 4-μm thick serial sections were cut on a MICROM HM355s rotary microtome (MICROM International GmbH, Walldorf, Germany) equipped with a waterfall based section transfer system (STS, MICROM). The cross-sections were placed on histological blades treated with poly-L-Lysine (Sigma-Aldrich, Munich, Germany). After Hematoxylin–Eosin classical staining, cross-sections were evaluated and photographed using a Nikon Eclipse 55*i* light microscope equipped with a Nikon DS–*Fi*1 CCD high definition video camera (Nikon Instruments, Apidrag, Bucharest, Romania). Images were captured, stored and analyzed using Image ProPlus 7 AMS software (Media Cybernetics Inc., Marlow, Buckinghamshire, UK) [33,34].

#### 3.2.8. Antimicrobial and Anti-Adherence Assay

*S. aureus* ATCC 25923 and *E. coli* ATCC 25922 were purchased from the American Type Cell Culture (ATCC). For establishing the minimal inhibitory concentration (MIC) of the tested nanoparticles, two-fold microdilutions of nanoparticles suspensions prepared in sterile saline buffer were done in nutrient broth previously distributed in 96 multi-well plates. Each sample was inoculated with 10 μL from *S. aureus* or *E. coli* microbial suspensions of a density corresponding to 0.5 McFarland (~10^8^ bacterial cells). Negative and positive controls were used. The plates were incubated for 24 h at 37 °C, in order to establish the influence of different concentrations of nanoparticles solutions on the planktonic cells growth. The MICs were considered as the last dilution of each tested compound which inhibited the microbial growth.

Microbial adherence in the presence of fabricated magnetite nanostructures was analyzed in 6 multi-well plates (Nunc, Invitrogen, Bucharest, Romania). Different dilutions of the tested suspension containing fabricated nanoparticles were distributed in 96 well plates. 200 µL of *S. aureus* or *E. coli* inoculum with standard density of 1–3 × 10^4^ CFU/mL were added in each well. Samples were incubated for 24 h at 37 °C. Adherence ability was analyzed after 24 h by crystal violet assay [35] the absorbance of the stained samples after acetic acid release was measured at 490 nm using a spectrophotometer.

## 4. Conclusions

This paper reports the successful fabrication of a biocompatible Fe_3_O_4_@AMO nanosystem, with great antimicrobial activity. This bio-active nanosized material proved to enhance the efficacy of low doses of amoxicillin against both the Gram negative pathogen *E. coli* and the Gram positive *S. aureus*. Furthermore, the obtained functionalized magnetite was proved to be well circulated through the mammalian body, offering the perspective of being used as an efficient drug delivery and controlled release nanosystem for active drugs in different localized infections.
